# Dermoscopy-assisted prevalence of hair loss after COVID-19 vaccination among an Egyptian population: a cross-sectional study

**DOI:** 10.1007/s11845-023-03493-5

**Published:** 2023-08-15

**Authors:** Amr M. Ammar, Ibrahim S. Ibrahim, Abbas N. Mohamed, Mohamed L. Elsaie

**Affiliations:** 1 Department of Dermatology, Venereology and Andrology, Al-Azhar University, Cairo, Egypt; 2 Department of Dermatology, Venereology and Andrology, Medical Research and Clinical Studies Institute, National Research Centre, Giza, Egypt

**Keywords:** Alopecia, Telogen effluvium, Trichoscopy

## Abstract

**Background:**

Coronavirus disease (COVID-19) currently named SARS-CoV-2 is a contagious disease caused by a coronavirus; incompatible data are present on the possible relationship among COVID-19 vaccines and hair loss.

**Aims:**

The objective of the current study was to assess dermoscopically the prevalence of hair loss among an Egyptian population following COVID-19 vaccination.

**Methods:**

A total of 2000 participants were enrolled in this cross-sectional study. Adult males and females who received one of recognized COVID-19 vaccine were included, irrespective of the status of previous COVID-19 infection. Those who were aged less than 18 years or above 60 years were excluded. Furthermore, subjects self-reporting hair loss were assessed by dermoscopy.

**Results:**

Among the studied cases, n = 478 (23.9%) complained of hair loss following vaccination. The majority of cases noticed their hair loss during the first 2 months post-vaccination (n = 215 after the first month and n = 158 after the 2nd month respectively).

**Conclusion:**

We reported prevalence of post-vaccination hair fall that was confirmed by trichoscopy and which affected approximately one quarter of participants who received COVID-19 vaccines. Other factors, such as stress and infection, cannot be excluded and remain to be further investigated by larger multicenter studies.

## Background

A number of skin conditions and reactions have been reported after COVID-19 vaccinations. A number of skin local reactions such as swelling, induration, redness, and pruritis as well as injection site pain and tenderness were among the commonly observed following vaccinations. Other reported manifestations included acute type I hypersensitivity reactions such as angioedema, urticaria, and anaphylaxis as well as delayed type IV hypersensitivity reactions and autoimmune-mediated cutaneous lesions such as vasculitis [1].

Hair loss accompanying (COVID-19) infection as well as its various vaccine strains has been of much concern. No clear understanding of the mechanism behind vaccine-related hair loss had been explained; however, a number of factors were hypothesized [2]. COVID-19-infected individuals had been more prone to microthrombi formation that caused compromise of the vascular circulation within scalp hair follicles [2].

Dermoscopy is a tool that is able to magnify anatomic and morphologic structures not clear enough to the naked eyes. Within hair follicles, dermoscopy allows better visualization of perifollicular and inter follicular patterns as well as alteration in hair shaft thickness and density.

Trichoscopy utilizes dermoscope for diagnosing and assessing hair disorders and was first used as a term in 2006. The hand-held, in-office easy to use tool allows for rapid non-invasive assessment of inflammatory hair disorders [3].

The objective of the current study was to assess dermoscopically the prevalence of hair loss among an Egyptian population following COVID-19 vaccination.

## Methods

This was a multicenter, cross-sectional study conducted from January 2022 till January of 2023. A total 2030 participants attending dermatology clinics at university as well as ministry of health hospitals were included in the study after their consenting. Adult males and females who received one of recognized COVID-19 vaccine were included, irrespective of the status of previous COVID-19 infection. Those who were aged less than 20 years or above 60 years were excluded. Out of the 2030 participants who were primarily included, 30 subjects were later excluded leaving 2000 participants for data analysis. Exclusions were for lack of evident COVID-19 vaccination certification or for failure to show up following agreement to participate.

## All participants were subjected to

Data collection using a questionnaire covering the following characteristics: age, gender, hair fall before and after COVID-19 vaccines, type of vaccine, previous history of COVID-19 infection, and preexisting conditions that might be related to hair fall. A pilot study was conducted on 20 volunteers to ensure clarity and convenience of the questions and to estimate the time needed to fill the questionnaire. No identifier or sensitive data were collected. Those complaining of hair loss were subjected to complete general and local hair examination using trichoscopy to confirm hair loss. Dermoscopic evaluation (DermLite DL4) and imaging were reproduced at baseline, 3 months, and 6 months for follow-up.

The study was conducted in accordance with the Declaration of Helsinki and upon approval by the Local Ethical Committee—Al Azhar University N: 000084. Statistical analysis using continuous variables was presented as mean ± SD (standard deviation) for parametric data and median (min–max) for non-parametric data. Qualitative data were described using number and percent. Association between categorical variables was tested using chi-square test. For all statistical tests, *p*-value > 0.05 was considered not significant and *p*-value < 0.05 was considered significant. All data were analyzed using IBM SPSS Statistics for Windows, Version 26, and Microsoft Excel 365.

## Results

The mean age of studied population was 30.77(± 7.54 SD) with range 21–54 years. No age significance was demonstrated between males and females *p* = 0.74. Among the studied cases, there were 524 who received Astra Zeneca vaccine; of whom 248 cases (47.3%) were females and 276 cases (52.7%) were males. Of the 32 cases who received Johnson and Johnson vaccine, 8 cases (25.0%) were females and 24 cases (75.0%) were males. Sixty-eight (68) subjects reported receiving Pfizer-BioNTech COVID-19 vaccine and twelve (12) received Moderna COVID-19 vaccine. Among all participants, 23.8% (*n* = 476) received Sinopharm vaccine and among them 200 were males and 276 were females. Sinovac and Sputnik vaccines were administered by 416 and 380 cases respectively. Of all 2000 cases reported in this study, 92 confirmed vaccination with Sputnik Light vaccine, among which 13 were females and 79 were males. Among included subjects, 98% (*n* = 1960) were not diagnosed with COVID-19, while only 40 cases (2%) reported positive infection with COVID-19.

Among the studied cases, *n* = 478 (23.9%) complained of hair loss following vaccination. The majority of cases noticed their hair loss during the first 2 months post-vaccination (*n* = 215 after the first month and *n* = 158 after the 2^nd^ month respectively) (Tables 1 and 2). Of the 478 cases complaining of hair loss, 389 of them presented with acute onset telogen effluvium (TE), 13 reported acute exacerbations of their androgenetic alopecia, 40 presented with new onset alopecia areata, and 20 experienced recurrences in preciously treated alopecia areata (Tables 2, 3, 4, and 5).

**Table 1:** Distribution of the studied cases according to sex and type of vaccine

**Vaccine**	**Study group (*n*** **= 2000)**			***P*-value**
	**Number**	**Female**	**Male**	
**AstraZeneca**	*n* = 524	248 (47.3%)	276 (52.7%)	
**Johnson and Johnson**	*n* = 32	8 (25%)	24 (75%)	
**Moderna**	*n* = 12	1 (8.3%)	11 (91.7%)	
**Pfizer**	*n* = 68	12 (17.6%)	56 (82.4%)	*X*^2^ = 96.11
**Sinopharm**	*n* = 476	200 (42%)	276 (58%)	*P-value < 0.001*
**Sinovac**	*n* = 416	144 (34.6%)	272 (65.4%)	
**Sputnik**	*n* = 380	208 (54.7%)	172 (45.3%)	
**Sputnik light**	*n* = 92	13 (14.1%)	79 (85.9%)	

*X*^2^: chi-square test. *P*-value < 0.05 is considered significant

**Table 2:** Prevalence and frequency of hair loss among studied subjects in relation to type of vaccine used

**Vaccine**	**Hair loss**	**Study group (*n*** **= 2000)**		***P*-value**
		**Frequency**	**Percent**	
**AstraZeneca** ***n* = 524**	No	360	68.7%	
	Yes	164	31.3%	
**Johnson and Johnson** ***n*** **= 32**	No	28	87.5%	
	Yes	4	12.5%	
**Moderna** ***n*** **= 12**	No	10	83.3%	
	Yes	2	16.7%	
**Pfizer** ***n* = 68**	No	56	82.4%	
	Yes	12	17.6%	X^*2*^* = 28.42*
**Sinopharm** ***n*** **= 476**	No	360	75.6%	*P < 0.001*
	Yes	116	24.4%	
**Sinovac** ***n*** **= 416**	No	324	77.9%	
	Yes	92	22.1%	
**Sputnik** ***n* = 380**	No	312	82.1%	
	Yes	68	17.9%	
**Sputnik light** ***n* = 92**	No	72	78.3%	
	Yes	20	21.7%	

*X*^2^: chi-square test. *P*-value < 0.05 is considered significant

**Table 3:** Frequency and percentage of hair loss after vaccination

**Hair loss**	**Study group (*n*** **= 2000)**	
	**Frequency**	**Percent**
**1**^**st**^** month**	215	45.0%
**2**^**nd**^** month**	158	33.1%
**3**^**rd**^** month**	45	9.4%
**4**^**th**^** month**	36	7.5%
**5**^**th**^** month**	24	5.0%
**Total**	478	100%

**Table 4:** Self-reported onset of hair loss following vaccination

**Vaccine type**	**Study group** **(*n *= 478)**	**1**^**st**^ **month**	**2**^**nd**^ **month**	**3**^**rd**^ **month**	**4**^**th**^ **month**	**5**^**th**^ **month**
**AstraZeneca**	*n* = 164	64	72	16	8	4
		39.0%	43.9%	9.8%	4.9%	2.4%
**Johnson and Johnson**	*n* = 4	0	4	0	0	0
		0.0%	100%	0.0%	0.0%	0.0%
**Moderna**	*n* = 2	0	2	0	0	0
		0.0%	100%	0.0%	0.0%	0.0%
**Pfizer**	*n* = 12	8	0	0	4	0
		66.7%	0.0%	0.0%	33.3%	0.0%
**Sinopharm**	*n* = 116	35	48	13	12	8
		30.2%	41.4%	11.2%	10.3%	6.9%
**Sinovac**	*n* = 92	32	32	8	8	12
		34.8%	34.8%	8.7%	8.7%	13.0%
**Sputnik**	*n* = 68	56	0	8	4	0
		82.4%	0.0%	11.8%	5.9%	0.0%
**Sputnik light**	*n* = 20	20	0	0	0	0
		100%	0.0%	0.0%	0.0%	0.0%
**Chi-square test**		*X*^2^ = 137.4, *P*-value < 0.001				

*X*^2^: chi-square test. *P*-value < 0.05 is considered significant

**Table 5:** Patterns of hair loss among COVID-19 vaccinated individuals

**Vaccine**	**Study group (*n*** **= 478)**				
	**Acute TE**	**Exacerbated AGA**	**New onset A.A**	**Overlap AGA and TE**	**Recurrent A.A**
**Astrazeneca** *n* = 164	139 (84.8%)	1 (0.6%)	12 (7.3%)	8 (4.9%)	4 (2.4%)
**Johnson and Johnson** *n* = 4	4 (100%)	0	0	0	0
**Moderna** *n* = 2	2 (100%)	0	0	0	0
**Pfizer** *n* = 12	4 (33.3%)	0	4 (33.3%)	4 (33.3%)	0
**Sinopharm** *n* = 116	96 (82.8%)	4 (3.4%)	8 (6.9%)	0	8 (6.9%)
**Sinovac** *n* = 92	72 (78.3%)	4 (4.3%)	12 (13.0%)	0	4 (4.3%)
**Sputnik** *n* = 68	52 (76.5%)	4 (5.9%)	4 (5.9%)	4 (5.9%)	4 (5.9%)
**Sputnik light** *n* = 20	20 (100.0%)	0	0	0	0
**Total**	389	13	40	16	20
**Chi-square test**	*X*^2^ = 74.7, *P*-value < 0.001				

*TE *telogen effluvium, *AGA *androgentic alopecia, *A.A *alopecia areata

*X*^2^: chi-square test. *P*-value < 0.05 is considered significant

**Fig. 1:**
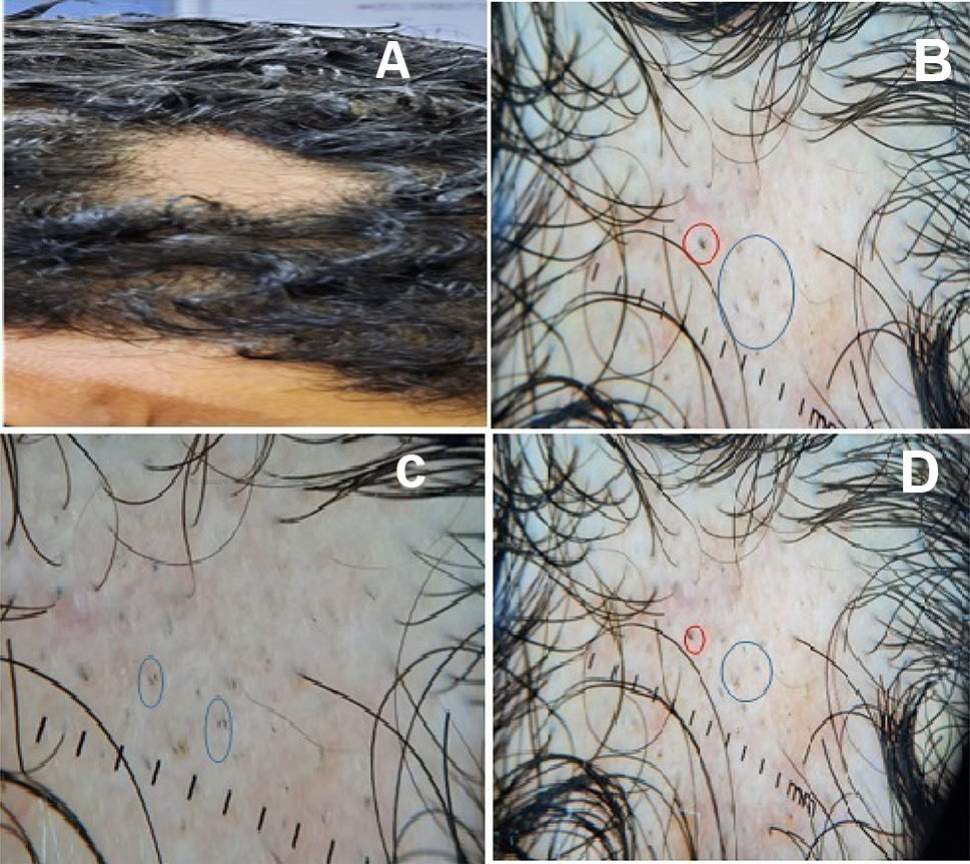
30-year-old male complaining of localized hair loss of 3-month duration after 2^nd^ dose of vaccination. Clinical image (**A**) show localized shedding of hair in the frontal area of the scalp surrounded by preserved hair. Dermoscopy images (**B**–**D**) show presence of black dots (blue circles) and broken hairs (red circle) suggestive of alopecia areata

**Fig. 2:**
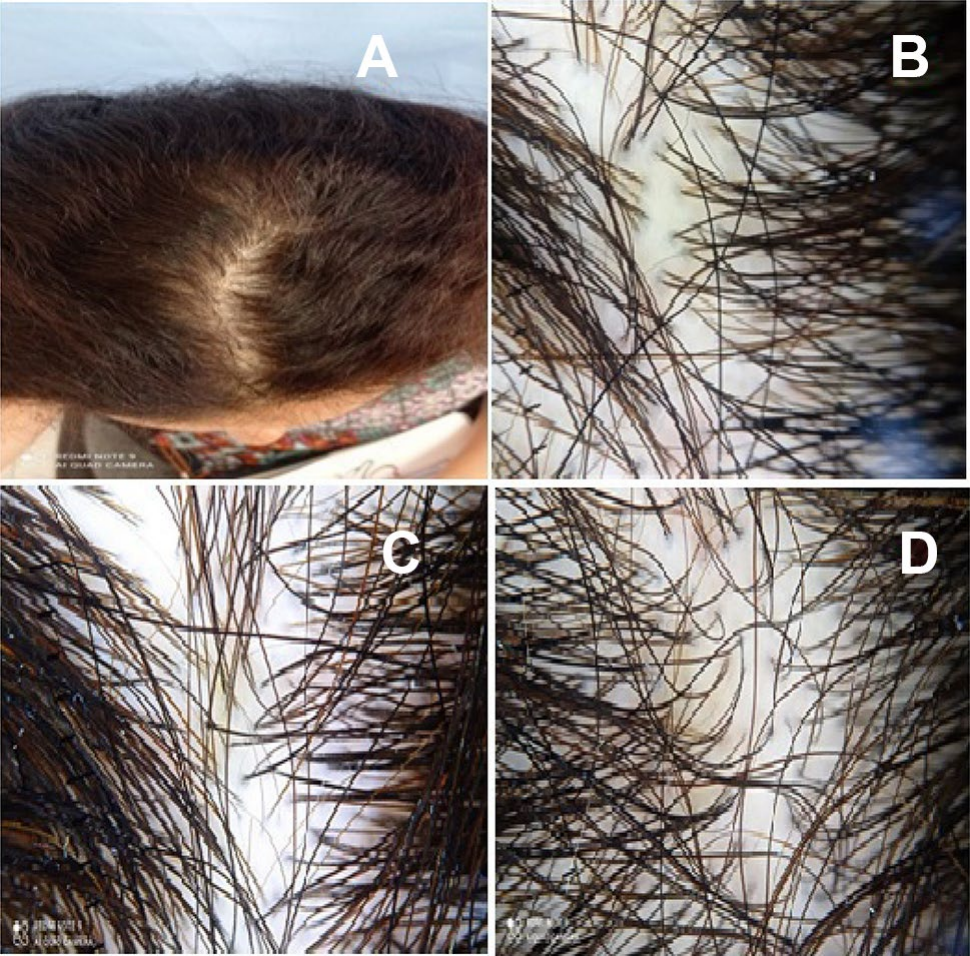
29-year-old female complaining of diffuse hair loss after 3 months of COVID-19 vaccination. **A** Clinical image showing diffuse shedding of hair in the frontal and middle areas of the scalp. Dermoscopy images (**B**–**D**) showing increased proportion of single hair follicle units and less than 20% hair diameter diversity suggestive of telogen effluvium

**Fig. 3:**
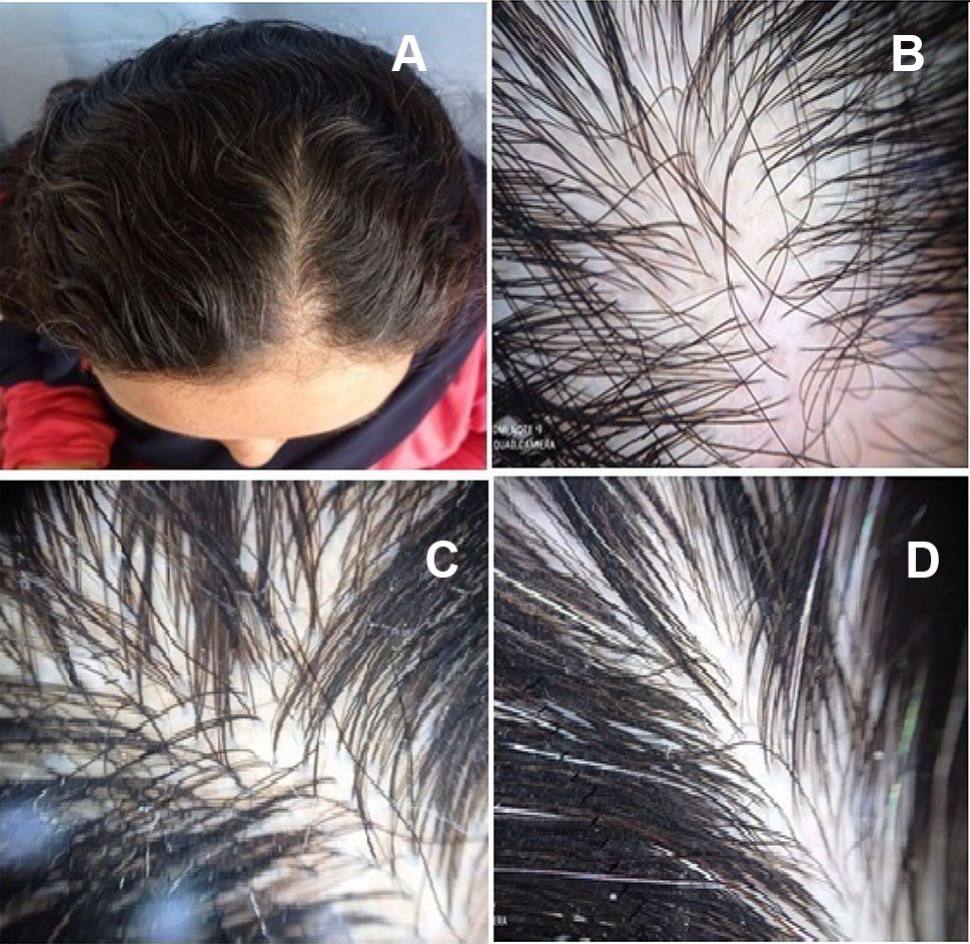
A 21-year-old female patient complaining of diffuse hair loss after 2 months of COVID-19 vaccination. **A** Clinical image showing diffuse shedding of hair in the frontal and middle areas of the scalp. Dermoscopy images (**B**–**D**) showing increased proportion of single hair follicle units and less than 20% hair diameter diversity suggestive of telogen effluvium

Significant associations between TE after COVID-19 vaccination and dose number exist (*p* < 0.05). Three hundred forty people (17%) reported TE after the first dose, while 138 (6.9%) reported TE after the second dose. Increased risk of TE is also noted across vaccine types with similar rates (84.8, 82.8, and 33.3% for Oxford/AstraZeneca, Sinopharm, and Pfizer-BioNTech vaccines, respectively). 98.7% of participants with post-vaccination hair loss reported recovery in hair growth or cessation of hair shedding by the 6^th^ month (Tables 4 and 5 and Figs. 1, 2, and 3).

## Discussion

Telogen effluvium–associated hair loss can last up to 6 months after proper treatment as well as removal of any triggering factors. Persistence of TE for more than 6 months implies chronicity of the condition and can be more challenging to treat. In this study, we found that approximately 23.9% of patients had diffuse hair loss for 6 months (acute TE) with the majority commencing 1 month post-vaccination (45.8%) and 33.2% after the second month.

Survey participants who received one or more vaccines against COVID-19 also had a statistically significant increase in TE. Cutaneous reactions after COVID-19 vaccination have been widely reported [4]. Local injection site reactions, urticarial eruptions, and morbilliform eruptions have been reported, but delayed; large local reactions are the most common cutaneous reactions [5].

Hair lost post COVID-19 vaccination was not widely discussed in the literature; however, a study from Poland demonstrated that only 2.2% of those vaccinated complained of acute hair loss of which 46.4% and 53.6% having hair loss after the first and second doses, respectively [6]. Another study from Saudi Arabia demonstrated hair loss after COVID vaccine to be as high as 63.2% with the majority after the first dose (55.8%) and 44.2% after the second dose [7]. Another study that assessed the prevalence of TE following COVID 19 vaccination demonstrated that out of 991 participants, 670 (67.6%) reported post-vaccination hair fall. The probable causes of post-vaccination hair fall were vaccine-related in 185 (27.6%) participants, other causes in 326 (48.7%) participants, and unclear in 326 (48.7%) participants [8].

The COVID-19 pandemic has increased the levels of anxiety and stress-related disorders among individuals which contributed to hair shedding and TE. Rates of acute hair shedding during the pandemic increased for which patients requested treatments to break such cycle. Interestingly, we found that only 24.4% of patients sought medical advice, similar to the rate of 18.9% reported by Turkmen et al. [9] and 22.9% reported by Alsalhi et al. [7].

Vaccines were identified to cross react with self-antigens leading to autoimmunity and to possibly trigger reactions in genetically susceptible individuals. It is possible that the messenger RNA SARS-CoV-2 Moderna and Pfizer vaccines can trigger a T cell–mediated immune response with the downstream effects of alopecia [10].

Alopecia areata (AA), characterized by patchy hair loss from the scalp, is an autoimmune condition precipitated by loss of immune privilege in the hair bulge leading to concentrates of natural killer (NK) cells within hair follicles [11, 12]. In the present study, new onset AA and recurrent AA were among the reported hair loss features among participants.

Hair loss following SARS-CoV-2 vaccination is an increasingly reported phenomenon. Newer reports are emerging with different forms of alopecias following COVID-19 vaccination [1]. Essam et al. also reported recurrent AA after a long period of disease quiescence in a middle-aged woman following immunization with COVID-19 vaccine [13].

Induction and rapid expression of type 1 interferon (IFN) against viral vaccines to promote the release of anti-chemokines such as interleukin 12 and interleukin 23 had been implicated in the development of AA. Moreover; this exaggerated production of IFNs can contribute to suppressing immunity and further promoting the development of AA. These findings suggest a potential role of vaccines to trigger autoimmunity, as observed in patients vaccinated for hepatitis B and hepatitis A that showed a significantly higher prevalence of alopecia areata [12].

To our knowledge, the current study is considered the first study to report post-vaccination hair fall among the public in Egypt, and probably the largest with 2000 participants included. Additionally, previous reports were mainly case reports with no ability to calculate prevalence or just questionnaire-based cross-sectional studies with no trichosopic confirmation of the diagnosis. Nevertheless, a number of limitations should be acknowledged such as absence of lab investigations and relying on self-reporting of patients as well as failure of follow-up of the condition for longer durations.

## Conclusion

In conclusion, we reported prevalence of post-vaccination hair fall that was confirmed by trichoscopy and which affected approximately one quarter of participants who received COVID-19 vaccines. Other factors, such as stress and infection, cannot be excluded and remain to be further investigated by larger multicenter studies.

## Data Availability

The data that support the findings of this study are available from the corresponding author upon reasonable request.

## Abbreviations

COVID-19: Coronavirus disease (COVID-19)
TE: Telogen effluvium
AA: Alopecia areata
NK cells: Natural killer cells
IFN: Interferon
